# Pre-hatching social interactions mediated by acoustic signals. Dynamics of click emission and hatching synchronization in birds

**DOI:** 10.1371/journal.pone.0330466

**Published:** 2025-09-03

**Authors:** Florencia Bazterrica, Juan Mateo Mayol, Estefano Vignetta, Vladimir Flores, Melina Rapacioli

**Affiliations:** Grupo Interdisciplinario de Biología Teórica, Instituto de Neurociencia Cognitiva y Traslacional (INCyT), Universidad Favaloro, INECO, CONICET, Buenos Aires, Argentina.; Ain Shams University Faculty of Agriculture, EGYPT

## Abstract

The present paper analyzes the sounds emitted by pre-hatching chicks, focusing on those named as “clicks,” which are thought to mediate pre-hatching social interactions and hatching synchronization. Representative acoustic signals were analyzed under three incubation conditions: (1) isolated pre-hatching chicks (n = 13), (2) pre-hatching chicks in contact with others of the same age (n = 14), and (3) pre-hatching chicks in contact with other of different age (n = 10 for each group: leader and follower). Customized MATLAB software was developed to (a) identify and isolate clicks from other recorded sounds, (b) represent them as temporal series of stochastic point processes, and (c) determine whether click emission dynamics resembled white noise or exhibited characteristics of informative signals. Mathematical methods were applied to analyze (a) temporal dynamics, (b) clustering patterns (via hierarchical clustering and log–log scaling), and (c) scaling properties (via power spectral density analysis) of clicks under each condition. The results reveal developmental-dependent changes in click temporal patterns. As hatching approaches, clicks evolve from isolated events to highly organized hierarchical clusters. Contacting chicks displayed greater temporal organization than isolated ones. Significantly, contact with more advanced chicks accelerated click dynamics in less developed embryos, while older embryos showed a slight delay, suggesting reciprocal social interactions. Spectral analysis revealed long-range correlations consistent with fractional Gaussian noise. These findings confirm that click sequences (a) exhibit physical characteristics of informative signals, (b) function as communication signals, and (c) align developmental processes among pre-hatching chicks. The study underscores the value of fractal analysis in describing physiological signals and expands our understanding of prenatal social interactions. The results suggest that acoustic signals may influence both hatching coordination and central nervous system development. This work provides insight into the evolutionary advantage of embryo communication and highlights the importance of studying how environmental disruptions may affect these critical prenatal processes.

## Introduction

Fertilization in birds is internal, and the embryonic development begins before oviposition. As the fertilized egg descends through the oviduct, the embryonic development progresses to the pregastrular blastoderm stage. Upon oviposition, the embryo enters diapause (a temperature-dependent state of latency) [1] that lasts until the hen initiates incubation. This process helps synchronize embryonic development ensuring that, despite being laid at different times, all embryos reach the same developmental stage at the onset of incubation, a phenomenon described as an evolutionary advantage [2]. In the poultry industry, this diapause plays a key role in egg storage [3].

The incubation period in chickens (Gallus gallus) lasts approximately 21 days. During this time, both the embryo and extraembryonic structures (amnion, chorion, allantois, and yolk sac) develop. Successful incubation requires controlled environmental conditions (temperature, humidity, air circulation) as well as characteristic egg movements. These conditions ensure proper heat and moisture exchange with the environment, promote nutrient absorption by the embryo and the expansion of the air chamber at the egg’s broader end [4]. In natural settings, the hen provides these conditions within the nest. An average clutch size in White Legorn hens typically ranges between 4 and 14 eggs, which remain in contact and are periodically repositioned by the hen [5–8]. Under artificial incubation, 3–24 periodic rotations are recommended between days 2–3 and 16 [9].

During incubation, the chick embryo changes its position within the egg. By day 18, the beak nears the air chamber, perforating the inner shell membrane between days 19 and 20 (internal pipping). Once the beak enters the air chamber, the chick begins to breathe. Approximately 7–12 hours later, the chick pierces the eggshell (external pipping), followed by hatching within 10–20 hours [9].

Pre-hatched birds of various species (ducks, gulls, quail, partridges, and others) produce different types of sounds [9]. The first sounds they emit result from beak movements and are known as beak “clapping”. Under natural conditions, once breathing begins, pre-hatched chicks interact with the hen through various species-specific calls, eliciting characteristic responses from the hens (e.g., changes in posture and temperature, movement of nest eggs, and emission of different call types). In turn, chicks adjust their calls and activity in response [10–12].

In addition to these calls, other sounds, known as “clicks”, are produced. Clicks are transmitted, by physical contact with neighboring eggs shells, to the adjacent chick embryos. Click emission begins with internal pipping and synchronizes with the respiration cycle. Clicks are considered to play a role in communication with neighboring pre-hatched chicks and contribute to hatching synchronization [13].

It has been reported that pre-hatched chicks of several species (domestic chicks, quail, ducks, and geese) adjust their click emission frequency to match that of neighboring embryos. As a result, adjacent chicks produce clicks at the same rate. In other words, embryos in physical proximity exhibit synchronized click rhythms [13–16].

The relationship between click signals and hatching synchronization was first proposed in quail. It was demonstrated that synchronization requires direct egg-to-egg contact, which reduces the hatching window by delaying more advanced embryos and accelerating less advanced ones [17–20]. Moreover, artificial clicks delivered at an optimal frequency of 3–6 clicks per second have been shown to accelerate hatching [15,21–23]. In chickens, physical contact between eggs also narrows the hatching window [14,24], and artificial click stimulation significantly promotes earlier hatching [16,25]. Other sounds -such as claps, calls, and music- have been ruled out as effective synchronizing stimuli [14]. There are two different phenomena designated in the literature by the same term: synchronization of clicks emission and synchronization of hatching. This phenomenon allows the less developed chicks to hatch simultaneously with those that are more advanced in their development.

In recent years, substantial progress has been made in understanding the role of sounds in embryonic development [26,27]. Hatching synchronization has been recognized as a widespread phenomenon among oviparous taxa. The role of physical contact between eggs in promoting synchronization has been documented in insects [28–31], amphibians [32,33], and reptiles [34–36]. In addition, several studies have identified specific signal properties that mediate this form of communication [28–30,32,33].

All available information in the literature regarding the “synchronizing” effect of clicks and also the generalized recognition that they serve a communication function indicates that they have an informative role. Besides, there are sonograms and frequency analyses that analyze clicks individually, as point acoustic phenomena [13]. The temporal evolution of individual clicks emission frequency (clicking rate) under synchronizing conditions and under a sound pressure level of 80 dB in laboratory incubators has also been described [13].

As far as we know, there are no studies in the literature about the informative nature that click temporal organization -dynamics of click emission- could have (temporal or hierarchical organization of clicks, existence or not of click trains, frequency of click trains, etc.). All these characteristics can be accurately analyzed by means of mathematical tools designed within the framework of theoretical models of signal analyses [37–41]. These methods allow a clear distinction between signal lacking information (such as white noise) and those that may contain information (stochastic processes with memory, with long-term correlations, fractality, etc.). In this context, one of the hypotheses of this work is that the click emission dynamics does not correspond to white noise -a random process that lacks informative value- but should correspond to some type of signal with memory, long-term dependency and hierarchical organization.

Furthermore, to our knowledge, there are no precise descriptions in the literature of the specific differences between sounds produced by pre-hatched chicks incubated in isolation versus those incubated in contact with other pre-hatched chick with the same or different time of incubation. We hypothesize that social context may modulate click dynamics, and that the dynamics of click emission differ significantly between embryos incubated in isolation and between those incubated in contact. The difference between both groups could be an indication of the existence of functionally significative inter-embryonic communication.

In sum, the present work aims at (a) analyzing the dynamics of clicks emission by means of standardized methods of signal analysis, its changes during the pre-hatching period and (b) analyzing the possible existence of click-mediated interactions between contiguous pre-hatched chicks and its potential effect on the dynamics of click emission and hatching acceleration.

## Materials and methods

### Experimental design and sample size

Seven incubation batches were conducted. Each began with 16 fertilized eggs collected within a short laying window of 2–3 days and similar weight. Embryo viability was assessed on day 17, and 8 viable eggs per batch were selected for audio recording. Eggs were assigned to one of the following experimental conditions:

- **Isolated pre-hatching chicks** (no physical contact): 13 valid recordings.
- **Contacting pre-hatching chicks of the same incubation age**: 14 valid recordings.
- **Contacting pre-hatching chicks with a 24-hour difference in incubation age**:
  - 10 leader embryos (older in asynchronous pairs) valid recordings
  - 10 follower embryos (younger in asynchronous pairs) valid recordings

A total of 112 fertile eggs were incubated, a total of 56 eggs were recorded, resulting in 47 usable audio recordings.

Details on incubation scheduling and egg allocation are provided in the S1 Appendix.

These experimental conditions were designed to either prevent or allow the transmission of clicks between embryos. In the isolated condition, eggs were incubated without any physical contact to avoid acoustic transmission through the eggshell. In the contacting condition, pairs of eggs were positioned in close contact, allowing potential click-mediated communication. In the asynchronous group, the 24-hour difference in developmental stage was used to assess the influence of early click exposure on less advanced embryos, as well as the potential reciprocal effect on more advanced ones. This developmental offset was chosen based on prior studies on hatching synchrony [16,18–20] and our own data showing that click emission sharply increases after 19.5 days (see Fig 14A).

**Fig 1:**
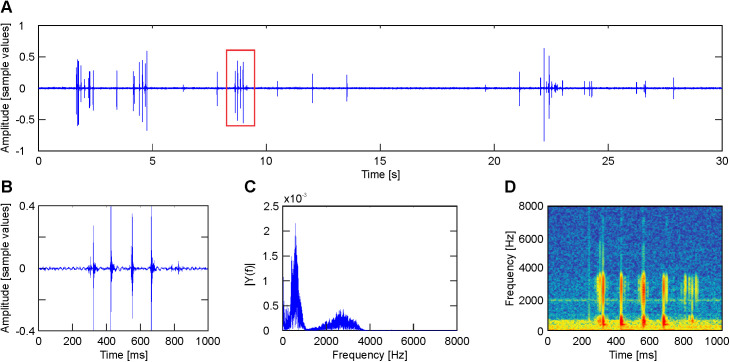
Characteristics of claps. A. Shows a 30-second-long segment of an audio signal made 32 hours before hatching. B. Magnification of the cluster indicated by the red box in Figure A. C. Fourier transform of the signal segment shown in Figure B. D. Spectrogram of the signal segment shown in B.

**Fig 2:**
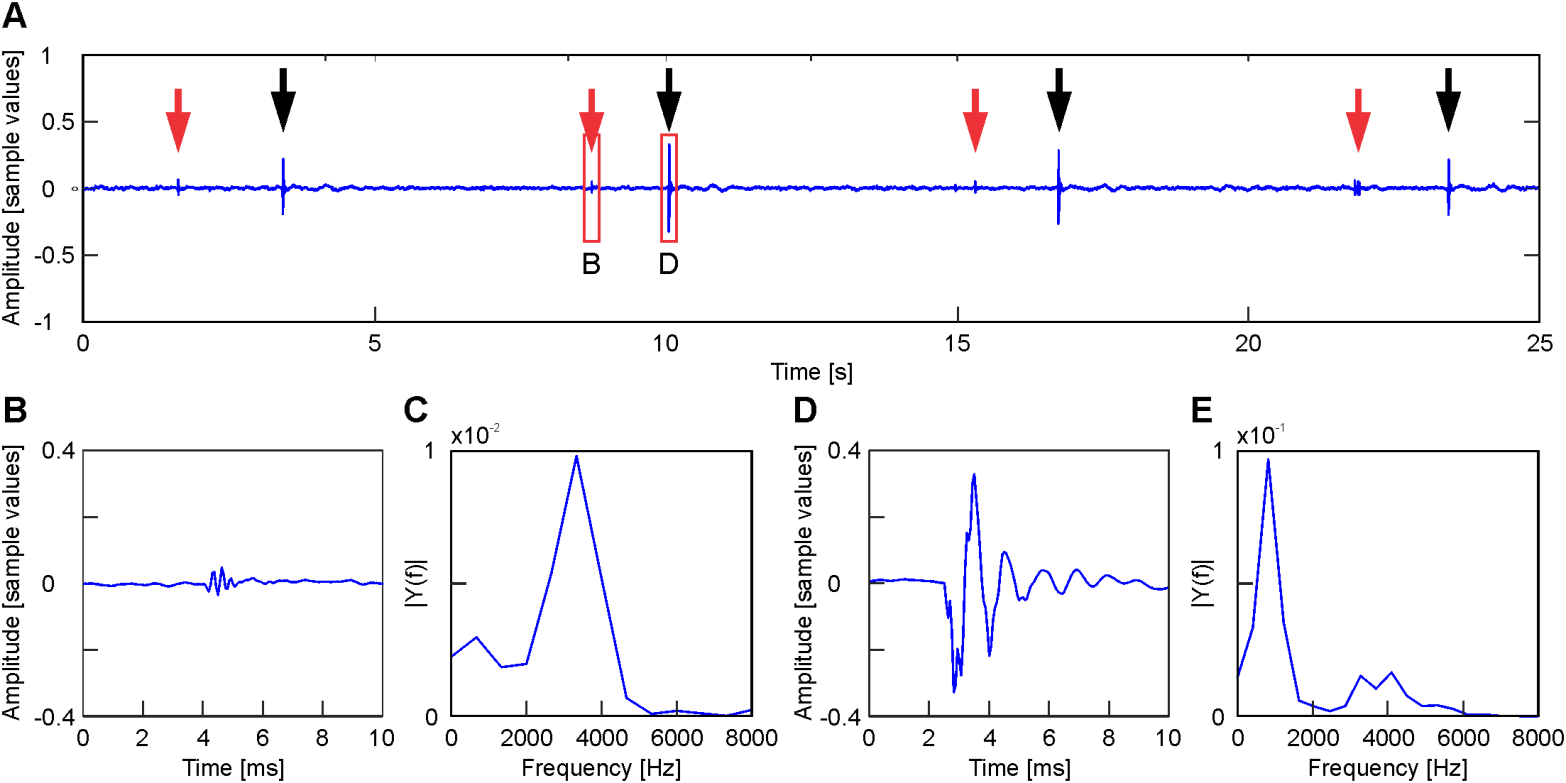
Inspiratory and Expiratory clicks. A. Shows a 25-second-long segment of a signal recorded 7 hours before hatching. Red arrows: inspiratory clicks; black arrows expiratory clicks. B and D. Zoom of the red boxes, B and D, indicated in Figure A, corresponding to inspiratory and expiratory clicks respectively. C and E. Fourier transform corresponding to inspiratory and expiratory clicks respectively.

**Fig 3:**
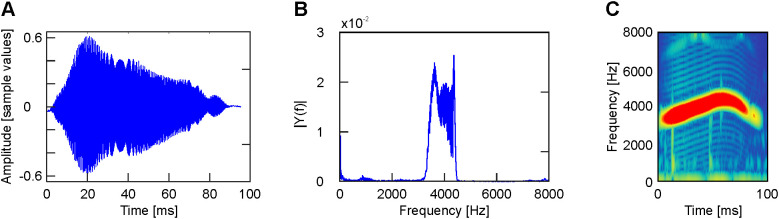
Example of a call. A. Temporal record. B. Fourier transform. C. Spectrogram. Record obtained 7 hours before hatching.

**Fig 4:**
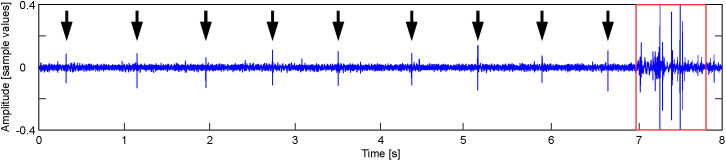
Movement noises. Shows an 8-second-long segment of a record obtained 30 minutes before hatching. The segment includes a regular succession of clicks (black arrows) and a set of fluctuations corresponding to body movements (red box).

**Fig 5:**
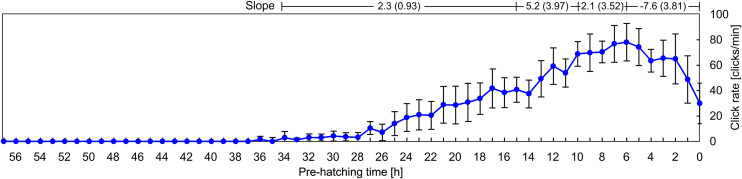
Variability in the click rate as a function of the pre-hatching time. Each point represents the mean ± standard deviation measured in 13 independents recordings. Slopes (rate of change in click rate) were calculated for four predefined pre-hatching time intervals: (a) from 34 to 15 pre-hatching hours, (b) from 15 to 10 pre-hatching hours, (c) from 10 to 6 pre-hatching hours, and (d) from 6 to 0 pre-hatching hours. For each interval, the slope is expressed as the median (interquartile range, IQR) of individual slopes. Statistical comparisons among the four intervals were performed using the Kruskal-Wallis test followed by Dunn’s post-hoc analysis. The results revealed statistically significant differences between successive intervals (p < 0.05), confirming that the rate of change in click emission differs significantly across the different phases of the pre-hatching period.

**Fig 6:**
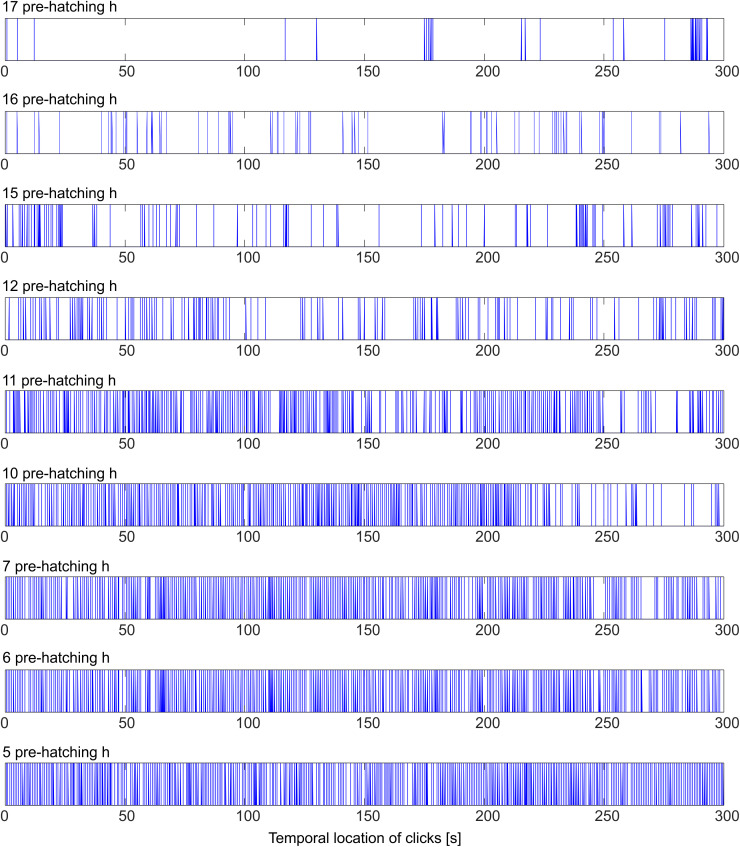
Surrogate binary signals. Three-hundred-second-long binary signals were constructed from audio signals obtained at different pre-hatching times (17, 16, 15, 12, 11, 10, 7, 6, 5 pre-hatching h). The bars (“1s”) indicate the temporal locations of clicks as a function of the time (s). Sub-series of “0s” between successive “1s”, which, in these binary signals, appear as empty spaces, represent the lengths of the inter-click intervals.

**Fig 7:**
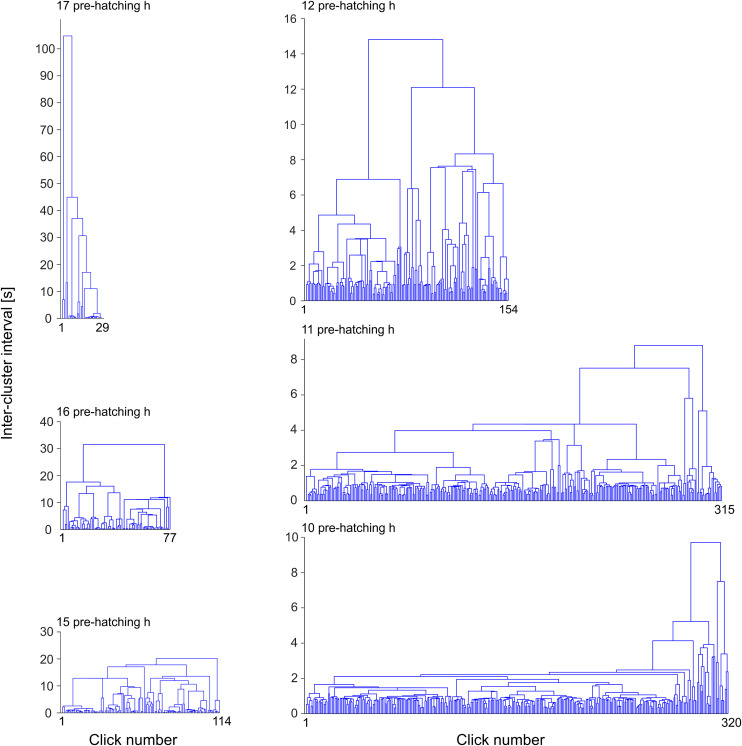
Hierarchical cluster analysis of binary signals. The figures represent dendrograms corresponding to binary signals obtained at different pre-hatching times (17 to 10 pre-hatching h). They reveal striking changes in the pattern of clusters organization during the pre-hatching stage. The height of the connectors (Π) represents inter-cluster intervals measured in s. The x axis represents the succession of clicks ordered by their time of appearance.

**Fig 8:**
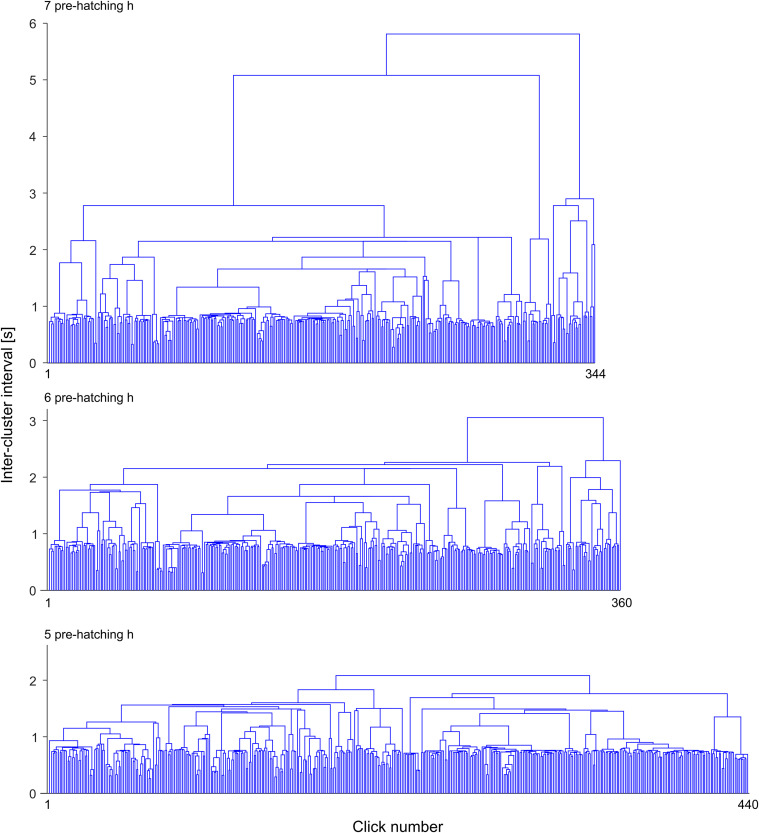
Hierarchical Cluster Analysis of binary signals. The figures represent dendrograms corresponding to binary signals obtained at different pre-hatching times (7, 6 and 5 pre-hatching h). They illustrate the changes in the pattern of clusters organization during the pre-hatching stage. The height of the connectors (Π) represents the inter-cluster intervals measured in s. The x axis represents the succession of clicks ordered by their time of appearance.

**Fig 9:**
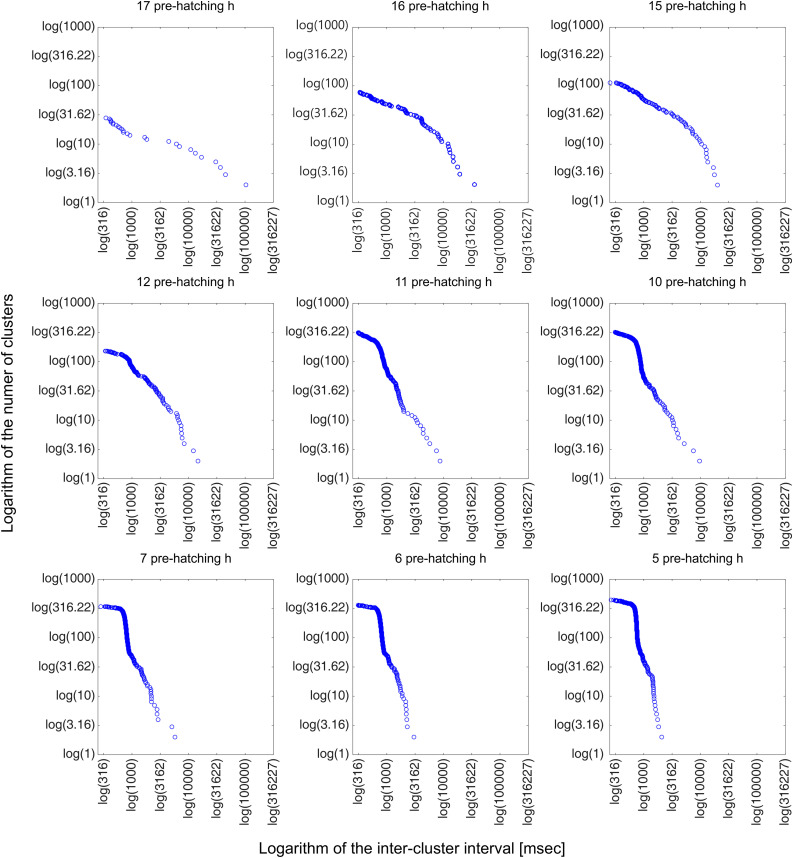
Bi-logarithmic plots of the number of click clusters as a function of the inter-cluster interval. Each plot shows the log–log relationship between the number of click clusters and the inter-cluster interval length, calculated at selected pre-hatching time points (17 to 5 pre-hatching hours). The data were derived from the dendrograms shown in Figs 7 and 8. The log-log plots show power law behaviors revealing the absence of a typical mean cluster size and that clicks are distributed in a continuous hierarchical set of clusters within clusters across scales. The figure shows that there is a typical variation in the clicks clustering as a function of the pre-hatching time. Detailed description in the text.

**Fig 10:**
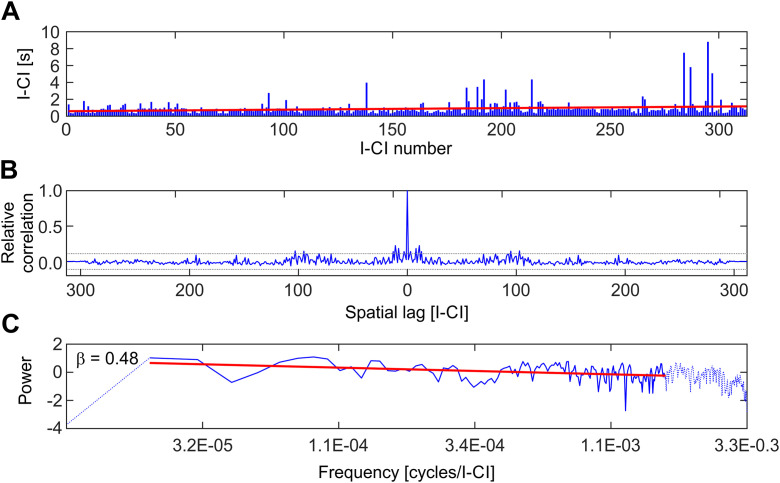
Frequency analysis of the inter-cluster interval (I-CI) signals. A. Illustrates an example of an I-CI signal obtained from an audio recorded at 11 h pre-hatching h. The red line was obtained by a third order polynomial fitting. B. Autocorrelation function (ACF) of the I-CI signal. The dotted lines indicate the 95% confidence interval. C. Fourier transform of the ACF. The slope of the line fitted by least squares linear regression is -β, where β is the scaling index of the process [33].

**Fig 11:**
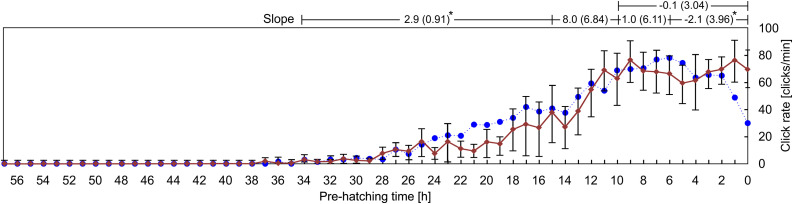
Evolution of the click rate as a function of the pre-hatching time. Each point represents the mean ± standard deviation of 14 individuals (7 groups, each composed of 2 contacting chicks). The blue dotted line represents the evolution observed in clicks emitted by isolated pre-hatching chicks (Fig 5). Slopes (rate of change in click rate) were calculated for five predefined pre-hatching time intervals: (a) 34−15 pre-hatching hours (phh), (b) 15−10 phh, (c) 10−6 phh, (d) 6−0 phh, and (e) 10−0 phh. For each interval, the slope is expressed as the median (interquartile range, IQR) of individual slopes. Statistical comparisons between intervals were performed using the Kruskal-Wallis test followed by Dunn’s post-hoc analysis. Significant differences were found between 34−15 phh and 15−10 phh, and between 15−10 phh and 10−6 phh (p < 0.05), but not between 10−6 phh and 6−0 phh. Additionally, slopes from the contacting group were compared with those from isolated embryos (Fig 5) using Mann-Whitney U tests. Significant differences (p < 0.05) were found during the intervals 34−15 phh and 6−0 phh, indicated by asterisks.

**Fig 12:**
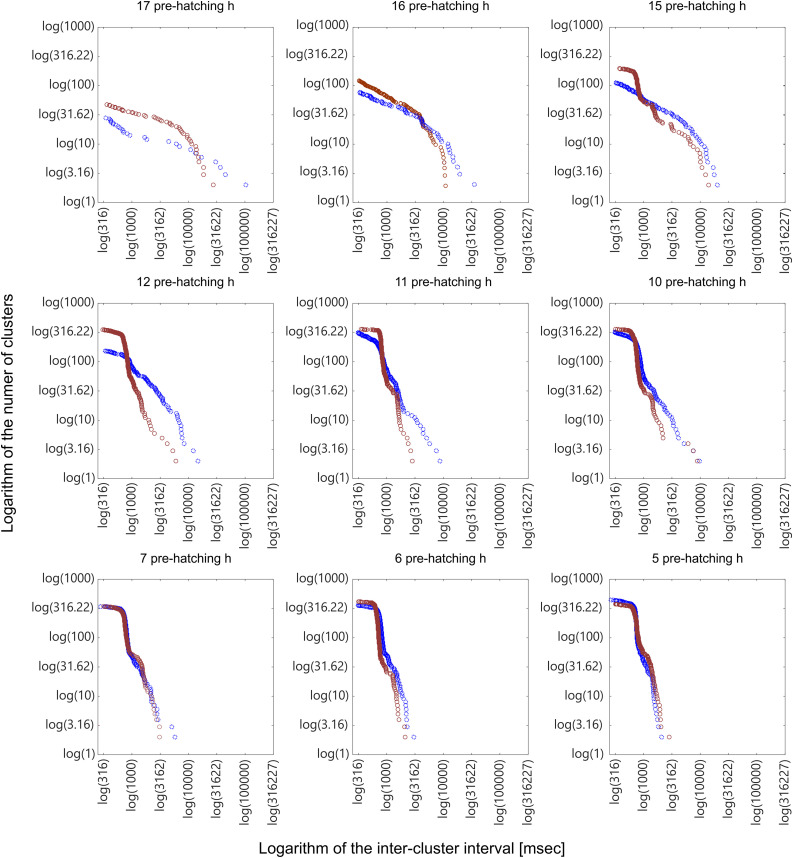
Bi-logarithmic plots of the number of clusters as a function of the inter-cluster interval length. The bi-logarithmic plots were obtained from dendrograms corresponding to 17, 16, 15, 12, 11, 10, 7, 6, 5 pre-hatching h. The plots show typical variations in the clicks clustering as a function of the pre-hatching time. Red circles: contacting pre-hatching chicks; Blue circles: isolated pre-hatching chicks. The dynamics of clicks clustering significantly differs between both groups. The changes in clustering dynamics observed in isolated chicks are anticipated in the groups of contacting chicks. See the description in the text.

**Fig 13:**
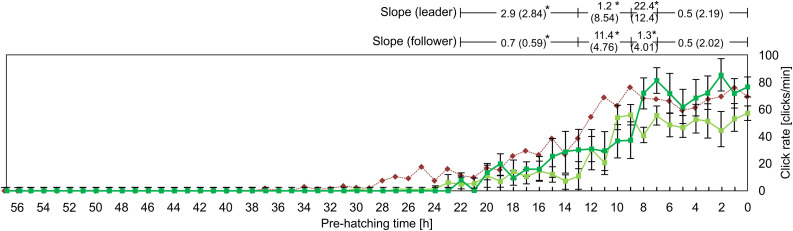
Evolution of the click rate as a function of the pre-hatching time. Each point represents the mean ± standard deviation of 10 experiments (10 leader chicks and 10 follower chicks). The red dotted line represents the evolution observed in contacting pre-hatching chicks (Fig 11). Slopes (rate of change in click rate) were calculated for four predefined pre-hatching time intervals: (a) from 22 to 13 pre-hatching hours (phh), (b) from 13 to 9 phh, (c) from 9 to 7 phh, and (d) from 7 to 0 phh, for both leader and follower groups. In the leader litter, the slope for the 9–7 phh interval was significantly higher than all other intervals (p < 0.05, Kruskal-Wallis test with Dunn’s post-hoc). In the follower litter, the slope for the 13–9 phh interval was significantly higher than all other intervals (p < 0.05, Kruskal-Wallis test with Dunn’s post-hoc). Comparisons between leader and follower litters revealed significant differences in the slopes for the intervals 22–13 phh, 13–9 phh, and 9–7 phh (p < 0.05, Mann-Whitney U test), indicated by asterisks.

**Fig 14:**
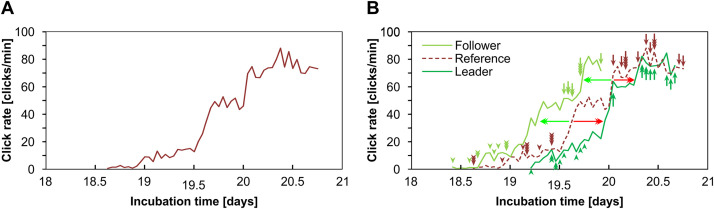
Evolution of the click rate as a function of the incubation time. A. Standard curve obtained by averaging the frequency of click emission of 14 independent experiments as a function of the incubation time. B. Shows the evolution of the click rate of the leader and follower litter (green lines). The reference curve (red dashed line) is also included. It is observed that the two asynchronous litters reciprocally influence each other. The leader litter undergoes a delay (horizontal red arrows) in respect to the reference curve and the follower litter shows an advance (horizontal green arrows) with respect to the reference. Arrowheads (upward and downward) indicate the onset of click emission for each individual recording, while arrows (upward and downward) indicate the hatching time, both color-coded according to group (follower, leader, or reference). Hatching time is defined here as the moment when the recording was interrupted due to intense noises produced by chick movements during hatching.

### Animals and incubation conditions

Animals were treated according to the Guide for the Care and Use of Laboratory Animals [42] and procedures were approved by the Laboratory Animal Care and Use Committee of Favaloro University.

Pathogen-free fertilized White leghorn chicken eggs were obtained from the Rosenbusch Institute (Buenos Aires). They were incubated at a temperature of 39°C and a relative humidity of 60%. Between days 2 and 17 of incubation, the eggs were rotated 24 times per day. Starting from day 17, the eggs were placed on an acoustic foam panel and organized according to the experimental groups (incubated in isolation or in contact with another egg).

In the asynchronous condition, part of the eggs was introduced into the incubator 24 hours after the initial group to create the 1-day developmental offset. Pairs of eggs were placed in individual soundproof boxes equipped with contact microphones. The microphone was attached to the air chamber region of each egg. Once the eggs were assigned to experimental groups, they were not moved until they hatched. These conditions resulted in similar hatching and survival rates across groups, with an average of approximately 80% in all experimental conditions.

### Signal acquisition and recording

For the acquisition and recording of the audio records of each egg, different devices were required to capture, amplify, digitize, record, and store the information of interest.

Audio signals were recorded for 5 minutes taken at one-hour intervals using contact microphones connected to preamplifiers and an 8-channel audio interface. The sampling rate was 16 kHz. Contact microphones were selected because they are highly sensitive to vibrations transmitted through solid media -such as the eggshell- while being minimally affected by ambient airborne noise. This property was essential for detecting egg-to-egg acoustic signals (clicks) while reducing environmental interference.

Further technical details regarding the hardware and configuration of the recording setup are available in the S2 Appendix.

### Signal processing

The obtained audio signals were processed using MATLAB® mathematical software. Various custom software applications were created in order to (a) suppress noise and eliminate non-specific sound events; (b) detect clicks; (c) eliminate transmitted clicks in contacting pre-hatching conditions and (d) click signals storage. As a final result of the processing of each audio signal, a text file was generated containing information about the location and amplitude of each click, the sampling frequency and the duration of the signal.

Details regarding each step of signal processing are available in the S3 Appendix.

For noise suppression and sound event filtering, a semi-automatic application using Daubechies 12 wavelet decomposition (6 levels) allowed the operator to reconstruct signals by selecting the details in which clicks were prominent, and noise was minimal. A detection threshold was manually adjusted for each signal to exclude background noise, typically set below 10% of the average amplitude of the reconstructed clicks, reflecting the substantial amplitude gap between click events and residual noise.

Click detection was carried out using the MATLAB® Find Peaks algorithm. While the amplitude threshold was manually adjusted for each signal, the MinimumPeakDistance parameter was fixed to ensure that each click was detected only once. This setting did not risk omitting neighboring clicks, as the average inter-click interval during high-rate periods (~120 clicks per minute) was approximately 125 times greater than the average click duration (500 ms vs. 4 ms).

For contacting embryos, a second software tool was used to identify and eliminate transmitted clicks. Based on time delay and amplitude differences between paired recordings, the software retained genuine clicks and discarded those transmitted from the neighboring egg.

Each processed signal was saved in a structured text file, containing time and amplitude information for each detected click, and formatted to support subsequent analyses.

### Mathematical analyses and statistical methods

Audio signals and different surrogate signals were analyzed by means of different mathematical methods in order to characterize the dynamics of click emission.

### Generation of surrogate signals

Different surrogate signals were generated from the click signals:

- Inter-click interval signals (I-CI). Correspond to the ordered sequence of time intervals, expressed in milliseconds, between consecutive clicks. The I-CI signal possesses a length corresponding to the number of clicks in the click signal.
- Binary signals. Represent the temporal location of clicks. The number “1” indicates the existence of a click. Sub-series of “0s” between successive “1s” represent the lengths of the inter-click intervals. The binary signal possesses the same length as the audio record.
- Click emission frequency signals. Correspond to the ordered sequence of click frequency (clicks/minute) during each recording hour. From these signals two types of average click emission frequency signals were generated: a) average click emission frequency as a function of the pre-hatching hour and b) average click emission frequency as a function of the incubation time.

Aiming at further characterizing the empirical I-CI signals, they were compared with two types of surrogate signals:

- Randomized (shuffled) I-CI signals. Correspond to signals generated by means of assigning new random positions to the intervals. The surrogate randomized I-CI signal possesses the same number of events, the same length, and the same probability density function as the original I-CI signal with a random internal correlation.
- Detrended signals. Correspond to signals generated by removing the global trend from the empirical I-CI signals. The result is a “detrended” signal with the same number of I-CIs and the same value of mean I-CI as the empirical signal. This procedure eliminates long-range correlations attributable to the nonstationary trend.

### Mathematical analyses

Several algorithms, implemented in *MATLAB* software, were used to estimate the dynamics of the different signals.

Audio signals were analyzed by means of Fourier transform and Spectrogram. Spectrograms were computed using a Hanning window of 320 samples at a sampling rate of 16 kHz, with a 25% overlap (80 samples). These parameters were selected to balance time and frequency resolution when visualizing the acoustic structure of claps and calls.

I-CI signals were analyzed by means of the power spectral density (PSD), i.e., the Fourier transform of the autocorrelation function. The PSD allows estimating the scaling exponent β of the signal and to distinguish between different models of stochastic processes: (a) stationary vs. nonstationary; (b) correlated (with long-term memory) vs. noncorrelated (white noise) vs. anti-correlated (short-term memory) processes.

Binary signals were analyzed by means of Hierarchical Cluster Analysis (HCA), which was used to detect and characterize the temporal organization of click emission. HCA was applied to these sequences to group temporally adjacent clicks into clusters based on inter-click intervals. The result is a dendrogram -a tree-like structure- where the horizontal axis represents the chronological order of clicks, and the vertical axis (height of branches) corresponds to the inter-cluster interval (the time between merged clusters). This analysis allows for the detection of hierarchical, multiscale organization in the temporal structure of click emission. The number of clusters obtained at different threshold values of inter-cluster interval was then plotted in log–log coordinates to identify power-law relationships, which are characteristic of fractal or self-similar patterns in time (clusters within clusters) that cannot be defined by a typical mean cluster size or by a typical mean inter-cluster interval length.

All these nonlinear methods have proven efficacy as mathematical tools to analyze and characterize the complexity of a signal [37–41,43–45].

### Statistical analysis

Descriptive statistics and comparisons amongst set of data were performed by means of the MATLAB Statistics Toolbox™. Statistical significance of the differences was determined by t test or Mann Whitney or by the one-way analysis of variance (ANOVA) or Kruskal-Wallis followed by multiple comparisons of means using Dunn’s post-hoc analysis. The value of p < 0.05 was considered as statistically significant.

## Results

### Characterization of the components of the audio signals

Several components, with well-defined characteristics, were identified in the audio signals.

1 **Claps** or beak-clapping. Each clap has an average duration of 14 ms. Claps are arranged in clusters of 4–5 successive claps, with a duration of about 0.6 s and are separated by intervals of 15–50 s (Fig 1A, B). Claps have a low-frequency band (0.3 to 1 kHz) of high amplitude and a high-frequency band (2–4 kHz) of low amplitude (Fig 1C, D). They disappear once the clicks are initiated.

2 **Clicks**. Two kinds of clicks -inspiratory (Fig 2A, B) and expiratory (Fig 2A, D) were identified.

Inspiratory clicks duration is about 1–2 ms and are composed of several 0.3 ms long fast oscillations and characterized by a band of frequency from 2 to 4 kHz (Fig 2B, C).

Expiratory clicks last about 3–5 ms and are composed of several 1 ms long oscillations and characterized by a broad band of frequency up to 5 kHz (Fig 2D, E). Only expiratory clicks were analyzed in this paper and, from here on they will be simply named as “clicks”.

3 **Calls**. Calls last about 100 ms with a frequency band ranging between 3 and 5 kHz (Fig 3).

4 **Noises produced by movement**. They are long-lasting noises -up to 1 s- composed of a set of high amplitude fluctuations (Fig 4) produced by strong body movements that contribute to the rupture of the covers of the shell and to the hatching.

### Isolated pre-hatching chicks

#### Variability in the frequency of clicks emission (click rate) as a function of the pre-hatching time.

Fig 5 shows that the production of clicks starts, with a very low rate, around 34 pre-hatching h. The click rate increases gradually until the 15 pre-hatching h. Then, a steeper increase takes place between the 15 and the 10 pre-hatching h. The click rate reaches a maximum -about 80–100 clicks/min- between 8 and 6 pre-hatching h and then decreases until hatching, when it oscillates around 30 click/min.

### Hierarchical cluster analysis

Hierarchical Cluster Analysis (HCA) was used to characterize the temporal organization of clicks as a function of the pre-hatching time. Surrogate binary signals, representing the temporal location of clicks, were generated as sequences of “1s” and “0s”. Each “1” corresponds to the temporal position of a click on the “x” (time) axis.

Fig 6 shows binary signals obtained from audio signals recorded at different pre-hatching times. Initially (17 pre-hatching h), the clicks appear isolated or as irregularly distributed clusters of short duration. Then, from 16 to 5 pre-hatching h, the number and length of clusters increases, and clusters of clusters appear. This implies that short neighboring clusters that are close to each other, are organized into larger clusters of higher hierarchy. This process of clicks clustering, an event that is difficult to describe qualitatively, can be analyzed quantitatively by means of the HCA (Figs 7–9).

Dendrograms displayed in Figs 7 and 8 were made from the binary signals shown in Fig 6. The “x” axis of the dendrograms represents the chronological order of clicks. The height of the connectors (Π) represented on the “y” axis correspond to inter-cluster interval length and, thus, they represent the time interval between two clusters that are included into a cluster of higher hierarchy.

It is clear from Figs 5 and 6 that the frequency of clicks emission (click rate) increases considerably as a function of the pre-hatching time. The HCA (Figs 7 and 8) shows that not only the frequency of clicks increases but there is also a notable increase in the complexity of the temporal organization of click emission. In order to show such an increase in the complexity more simply, bi-logarithmic representations of the evolution of the number of clusters as a function of the inter-cluster interval were constructed for each pre-hatching time.

Fig 9 illustrates that at 17 and 16 pre-hatching h, the bi-logarithmic relationship between the number of clusters and the inter-cluster interval fits a straight line. This fact implies that the clicks emission dynamics, from the beginning, is governed by a power law. However, the increase in the click rate that occurs after 15 pre-hatching hour involves a dynamic that becomes more complex since it does not fit a straight line. Rather, they seem to follow two different corresponding laws on two different time scales, i.e., there could be different power laws governing different time scales. This fact is revealed by the way in which points corresponding to different scales fit different slopes. Based on these considerations, subsets of points corresponding to different time scales were arbitrarily chosen to describe the entire process. Three inter-cluster interval scales ranges were defined: a) Inter-cluster intervals (I-CI) > 10 s, b) I-CI range between 10 and 1 s, and c) I-CI < 1 s. At 15 pre-hatching h, it is observed that the clusters separated by less than 10 seconds respond to a different clustering law than the one observed with the clustering of the clusters separated by a longer time. From the 12 pre-hatching h this change is observed on smaller scales. Clusters separated by less than 1 second have different dynamics than clusters separated by longer times. Note that the region corresponding to the scale inferior to 1 second has a typical slope between 12 and 10 pre-hatching h, which becomes horizontal later, from 7 pre-hatching h, remaining unchanged until the end of hatching. These characteristics can also be appreciated in the dendrograms illustrated in Fig 7. It can be observed that from 12 pre-hatching h, the number of clusters separated by less than 1 second increases.

### Characterization of signals as stochastic processes

Standardized methods of signal analysis were used to characterize the dynamics of clicks emission as a stochastic temporal process. These methods allow estimating the scaling index characteristic of the signal dynamics and identifying which kind of formal model an empirical signal fit.

The Power Spectral Density (PSD) was used to characterize the process in the frequency domain. In order to make illustrative comparisons, surrogate signals resulting from (a) the random mixing of clicks position and (b) the detrending of the original signals were used (see Materials and Methods).

Fig 10A–C shows the results of the autocorrelation function (ACF) and its Fourier transform (PSD) corresponding to I-CI signals obtained at the 11 pre-hatching h. The ACF shows that the autocorrelation remains over the upper limit of the confidence band for only 1 point (around 1 second). However, the correlation oscillates above and below the upper limit for approximately 12 seconds. The PSD reveals the existence of a power law between the amplitude and the frequency. Values of the scaling index β obtained from several experiments performed as a function of the pre-hatching time are presented in Table 1.

**Table 1:** Values of β (mean ± SD) estimated by PSD applied to I-CI signals and their corresponding surrogate signals at different pre-hatching h.

Pre- hatching h	Values of β (mean ± SD)		
	Original Signal	Randomized	Detrended
**20**	0.12 ± 0.08	0.02 ± 0.05	−0.04 ± 0.08
**11**	0.56 ± 0.22	0.01 ± 0.18	0.03 ± 0.07
**6**	0.38 ± 0.22	0.05 ± 0.12	0.06 ± 0.16
**2**	0.22 ± 0.10	0.06 ± 0.05	−0.07 ± 0.13

Table 1 Shows values of β obtained from signals recorded at 20, 11, 6 and 2 pre-hatching h in chicks incubated in isolation. The values of β are significantly > 0.0. These results indicate that they have power spectra of the type 1/f(β) and that the signals behave as realizations of a fractional Gaussian noise (fGn). The values of β obtained from both kinds of surrogate signals correspond, as it was expected, to white noise processes.

### Contacting pre-hatching chicks

When audio signals are recorded simultaneously on two contacting eggs, two different kinds of clicks -with different amplitude- are detected in each of them: (a) “own” clicks produced by each of them and (b) “transmitted” clicks emitted by the adjacent pre-hatching chicks. These two kinds of clicks can be easily identified, because of their different amplitudes, and can be filtered in order to analyze separately the “own” and the “transmitted” clicks (see Materials and Methods).

### Variability in the frequency of clicks emission (click rate) as a function of the pre-hatching time

Fig 11 shows the temporal evolution of the click rate on pre-hatching chicks incubated in contact. With comparative purposes, the evolution of clicks emission in isolated pre-hatching chicks is also represented -blue dotted line- in this figure.

It can be seen that in both cases, the emission of clicks starts approximately at the same time (about 34 pre-hatching h). Between 34 and 15 pre-hatching hours, both groups show a gradual and sustained increase in click rate; however, the rate of increase is slightly lower in the contacting group compared to isolated chicks (p < 0.05). Between 15 and 10 pre-hatching hours, a sharp increase is observed in both groups. Although the slope is steeper in the contacting group, the difference between groups in this interval was not statistically significant. Once the highest click rate value is reached by the 10 pre-hatching h, there is a plateau that, with small oscillations, persists until hatching. In fact, the group of contacting pre-hatching chicks does not undergo the typical decay of click emission observed before hatching in the isolated pre-hatching chicks (p < 0,05).

### Hierarchical cluster analysis

Dendrograms were constructed from binary signals obtained at the 17, 16, 15, 12, 11, 10, 7, 6, 5 pre-hatching h (not shown) and then the data were represented as bi-logarithmic plots of the number of clusters as a function of the inter-cluster interval length (Fig 12, red circles). For comparative purposes, the results obtained in isolated pre-hatching chicks (Fig 9) are also represented (blue circles) in Fig 12. It can be appreciated that there are significant quantitative differences between them that temporally coincide with those observed in the evolution of click rate (Fig 11).

Pre-hatching chicks in contact anticipate changes described in clicks emission dynamics for isolated chicks. The increase of the slope of clusters separated more than 10 s is already present at 17 pre-hatching h, two hours earlier. On the other hand, coinciding with the sharp increase in density of click emission observed between 15 and 10 pre-hatching h, the slope of clusters separated by less than 1 second starts to horizontalize at 15 pre-hatching h in contacting chicks, a phenomenon that occurs between 12 and 7 pre-hatching h in isolated chicks. From 10 to 5 pre-hatching h the distribution of points is similar in both groups.

### Characterization of signals as stochastic processes

Table 2 shows the results of frequency analyses -the ACF and its Fourier transform- performed on signals recorded at 20, 11, 6 and 2 pre-hatching h in contacting chicks. The values of the scaling index β estimated by the PSD are significantly > 0.0 (Table 2) indicating that the signals have power spectra of the type 1/f^(β)^. The table also shows that the values of β estimated in both kinds of surrogate signals correspond, as expected, to white noise processes. It is interesting that, during the early stages of click emission (20 and 11 pre-hatching h), there are statistically significant differences between the values of β corresponding to isolated and contacting pre-hatching chicks. These differences disappear immediately before hatching (6 and 2 h).

**Table 2:** Values of β (mean ± SD) estimated by PSD applied to ICI signals and their corresponding surrogate signals at different pre-hatching h.

Pre- hatching h	Values of β (mean ± SD)		
	Original Signal	Randomized	Detrended
**20**	0.57 ± 0.09*	0.01 ± 0.10	0.03 ± 0.03
**11**	0.20 ± 0.10*	0.04 ± 0.12	−0.06 ± 0.08
**6**	0.33 ± 0.21	0.02 ± 0.12	0.06 ± 0.16
**2**	0.33 ± 0.14	0.05 ± 0.10	0.09 ± 0.10

(*) indicate statistical differences with respect to the values of β estimated in corresponding signals recorded in isolated chicks (p < 0,05).

### Asynchronous (different ages) contacting pre-hatching chicks

In order to evaluate the possible effect of sensing external clicks on (a) the dynamics of clicks emission and (b) hatching acceleration, pre-hatching chicks of different developmental stages were incubated in contact. These asynchronous chicks, which were at different times of incubation, were chosen from two litters with a difference of 24 hours in incubation time. The more advanced litter, referred to as the “leader litter”, started incubation 24 h earlier than the less advanced “follower litter”.

### Variability in the frequency of clicks emission (click rate) as a function of the pre-hatching time

Fig 13 shows the evolution in the click rate in asynchronous-contacting pre-hatching chicks. The dark green and light green profiles correspond to the leader and follower litter, respectively. For comparative purposes, the evolution of the frequency of clicks emission of contacting pre-hatching chicks (red profile) was also included in this figure.

In both cases (leader and follower litters), the emission of clicks starts around the 24 pre-hatching h. A time significantly delayed, i.e., near hatching, compared to that observed in the group of isolated and contacting pre-hatching chicks (around 34 pre-hatching h). During the following hours the click rate in the leader litter increases gradually and, after a sharp increase between 9 and 7 pre-hatching h, reaches a maximum -around 80 clicks/min- by the 6 pre-hatching h and then this rate is maintained, with oscillations, until hatching.

There are some differences between the leader and the follower litters: a) the click rate performed by the follower litter is very low between 24 and 13 pre-hatching h; b) between the 13 and 9 pre-hatching h, the click rate increases sharply, reaches the maximum (60–70 clicks/min) and then, similarly to the leader litter, remains unmodified, with minor oscillations, until hatching.

### Effect of click-mediated stimulation on pre-hatching chicks of different ages incubated in contact

In order to analyze the possible accelerating effect of clicks produced by the leader litters on the development of the follower ones, reference standard curves were constructed using the time of incubation -instead of the pre-hatching time- as the independent variable (Fig 14). For comparative purposes, the evolution in the click rate observed in the group of contacting pre-hatching chicks (Fig 14A) was constructed. This curve was used as reference to evaluate the changes in the click rate that take place in the groups of asynchronous contacting pre-hatching chicks.

Fig 14A shows that the standard curve displays two typical characteristics that can be advantageously used to make simple and clear comparisons. In fact, it shows sharp increases in the click rate in two distinct moments: a) a first sharp increase on day 19.5, which brings the click rate to about 50 clicks/min, and b) a second one that takes place on day 20, with this second increment the click rate reaches a maximum of about 85 clicks/min. This value continues, with oscillations, until hatching (median: 20.3 days; range: 20.04–20.79 days).

Fig 14B shows profiles of mean values of click rate recorded in leader and follower litters. In theory, it should be expected that, in the absence of any influence between them, the profiles of frequency of click emissions should be similar in both groups, and similar to the reference curve (Fig 14A). However, this is not the case. Instead, both groups of eggs modify their behavior. The chicks of the leader litter show a delay in the first increment (“a”) of about 10 h and a delay in the second increment (“b”) of about 6 h (red arrows) and the hatching occurs within the expected time (median: 20.4 days; range: 20.04–20.7 days). By contrast, the chicks of the follower litters undergo an advancement in the first increment (“a”) of about 9 h, an advance in the second increment (“b”) of about 12 h (green arrows) and, also, an advance in hatching time (median: 19.7 days; range: 19.5–19.9 days).

## Discussion

The results presented in this study provide a detailed characterization of the sounds emitted by pre-hatching chicks, with an emphasis on those designated as “clicks” which are synchronized with respiratory movements. Developmental changes in the dynamics of clicks emission and their temporal organizations were analyzed under different incubation conditions, including isolation, contact with eggs of the same age, and contact with eggs of different ages. The findings confirm and expand previous concepts regarding pre-hatching social communication in birds and suggest that acoustic interactions between embryos may influence the timing of hatching, potentially contributing to coordinated developmental trajectories.

### Signal characterization

The distinction between several acoustic components (clicks, beak claps, and calls) supports the functional complexity of sound emissions in pre-hatching chicks. In particular, the clicks exhibit a distinctive developmentally regulated pattern of emission that characteristically changes as a function of the time of incubation. The transition from the simplest patterns to those more complex clustered patterns suggests a progressive increase in temporal organization. This transition is likely dependent of mechanisms involved in the development of the central nervous system.

The power spectral density (PSD) analysis demonstrates that the signals representing the temporal dynamics of click emission exhibit characteristics typical of fractional Gaussian noise (fGn). This characterization as a stochastic stationary process highlights the complex, autocorrelated nature of these signals. Hierarchical cluster analyses allow characterizing three inter-cluster interval scales with different dynamics: a) Inter-cluster intervals (I-CI) > 10 s, b) I-CI ranging between 10 and 1 s, and c) I-CI < 1 s. Two of these scales (b and c) coincides with those during which correlation remains over the confidence interval.

These observations align with an increasing body of evidence that reinforces the usefulness of mathematical fractal analyses in more reliably describing and characterizing the dynamics of signals that appropriately represent the operation of complex physiological processes [33–37,39–41], emphasizing the utility of these tools in characterizing the complexity of biological processes.

### Effects of egg contact

The analysis of click emission in contacting pre-hatching chicks revealed distinct temporal dynamics compared to isolated embryos. Although both groups exhibited similar onset times for click emission, the contacting group showed a significantly slower initial increase in click rate, followed by a steep rise in both groups. Notably, after reaching the peak, contacting embryos maintained high click emission levels until hatching, in contrast to isolated embryos, which showed a marked pre-hatching decline Additionally, PSD and hierarchical analysis reveal that the transition from a simple to a complex pattern is anticipated, suggesting that pre-hatching vibro-acoustic communication facilitates the development of more organized and robust emission patterns [18,27].

### Influence of asynchronous contact

Experiments with asynchronous contacting pre-hatching chicks highlight a bidirectional effect between the “leader” and “follower” groups. The leader litter experiences a delay in click emission frequency compared to the reference curve, while the follower litter shows a significant advance. It must be remarked that the follower litter displays a sharp increase between the 13 and 9 pre-hatching hours, this sudden increase is because the youngers need at least 15 hours of contact with a leader to experience the stimulating effect of the clicks emitted by pre-hatched chicks that are more advanced in development.

This suggests that clicks emitted by more developed eggs have an accelerating effect on less developed ones, while the former adjust their dynamics to maintain group synchrony. This phenomenon could plausibly be mediated by auditory mechanisms responding to external stimulation, potentially triggering neuroendocrine changes involving thyroid hormones [24,27,46]. While this hypothesis is not directly tested in the present study, it aligns with previous findings on the involvement of thyroid hormones in peri-hatching processes and central nervous system maturation. It has been observed that, during the peri-hatching period, thyroid hormones act in multiple processes, some of them are directly linked to hatching (such as the development of the musculature that is specifically used for the breakdown of the shell during hatching) and others are related to the transition to post-hatching life (such as lung maturation, metabolic and thermogenic transition and the development of the central nervous system) [46]. This last effect would be relevant given that, in agreement with previous papers [47], we have observed that chicks hatched prematurely (from younger eggs) are as mature as chicks hatched after incubation periods of normal duration, i.e., the degree of maturation of some functions of the central nervous system of the youngers chicks is quite similar to the degree of those hatched after an incubation of normal duration. This phenomenon is very significant because it does not only imply that the hatching process is being brought forward but it does also reveal a more general effect on the central nervous system development so that the degree of maturation of chicks of different incubation age is similar. Supporting this interpretation, preliminary data from our laboratory suggest that embryos exposed to click-mediated stimulation and undergoing precocious hatching exhibit elevated plasma T3 levels at hatching compared to controls [48,49]. This suggests that this phenomenon does not only correspond to a reflex, an event that involves low order functions, but also influences other superior areas that enable better global maturation of the CNS.

### Ecological and practical implications of hatching synchronization

Hatching synchronization is a widespread adaptive trait in precocial bird species, with both ecological significance and practical applications.

From an evolutionary perspective, synchronized hatching reduces brood vulnerability to predation by minimizing the time window during which chicks remain confined to the nest and exposed to predators [50]. This coordination ensures that all chicks leave the nest together with the parent bird, reducing the risk of predation and preventing late-hatching individuals from being abandoned [51]. In precocial species, both the parent and the brood typically depart from the nest within hours or days after hatching, moving together while alternating periods of movement, feeding, and brooding -behaviors that are closely linked to imprinting processes [9]. Coordinated hatching facilitates the immediate departure of the brood, enabling chicks to follow the hen and rapidly acquire essential survival skills such as foraging, predator avoidance, and social integration [52,53].

In natural conditions there are multiple processes that can promote hatching synchrony [54]. In artificial incubation, experimental studies have demonstrated that physical contact among eggs shortens incubation duration and reduces the variability in hatching time [14,50]. This finding has practical relevance for the poultry industry, where prolonged hatching windows -often extending up to 48 hours under artificial conditions compared to the typical 8–10 hours under natural brooding- are associated with compromised chick welfare, reduced flock uniformity, and impaired post-hatching growth due to delayed access to feed and water [14,50,55].

Several strategies have been explored to improve hatching synchrony in artificial incubation, including modifying egg arrangements to promote physical contact, providing auditory stimulation with maternal calls or embryo vocalizations, and optimizing environmental conditions [13,14,50,54–56]. Such approaches not only enhance chick quality and reduce economic losses but also offer promising avenues for improving animal welfare and refining incubation management practices.

Together, these ecological and applied perspectives underscore the relevance of hatching synchronization beyond basic developmental biology, highlighting its importance for both evolutionary ecology and commercial incubation systems.

### Implications and future perspectives

Our findings indicate that click emission is modulated by social context during the pre-hatching stage of development. The observed acceleration in click emission dynamics in embryos exposed to external acoustic stimuli -particularly those in contact with older embryos- suggests that chick embryos can adjust their behavior in response to the presence of conspecifics. This ability to modify click dynamics according to environmental and social conditions reflects a significant degree of neural plasticity during the pre-hatching period. It is also known that calls produced by the hen during the final stages of incubation act as external stimuli that influence embryonic behavior, reinforcing the idea that chicks are responsive to a variety of social acoustic cues even before hatching [9,57,58].

Future research is needed to explore the physiological and neurological mechanisms underlying this adaptation, the potential evolutionary advantages of such programming and the impact of these interactions on the evolution of social behavior during postnatal life.

It would also be relevant to examine how anthropogenic noise may disrupt these prenatal acoustic signals and affect development. Additionally, it would be relevant to analyze whether similar patterns are observed in other bird species or animals with comparable developmental strategies. The evaluation of the mechanisms that lead to the acceleration of development would allow progress in the study of an experimental therapeutic model that allows the development of the nervous system to be accelerated.

Overall, this work contributes to a deeper understanding of prenatal communication in birds and opens new lines of research on the influence of early social interactions on embryonic development.

## Supporting information

S1 Appendix Incubation chronology and experimental conditions. (PDF)

S2 Appendix Signal acquisition and recording. (PDF)

S3 Appendix Signal processing. (PDF)
