# Supplementary material for: Pre-hatching social interactions mediated by acoustic signals. Dynamics of click emission and hatching synchronization in birds
Source: PLoS One. 2025 Sep 3;20(9):e0330466. doi: 10.1371/journal.pone.0330466 (PMC12407395; doi:10.1371/journal.pone.0330466)
Supplement: S1 Appendix — (PDF) [file pone.0330466.s001.pdf]

S1 Appendix. Incubation chronology and experimental conditions

A total of seven incubation batches were carried out. Each incubation began with 16 fertilized eggs from the same clutch, selected to have similar weight. Embryo viability was assessed on day 17 of incubation, and 8 viable eggs were selected from each batch for recording.

- **Batches 1–3:**

  In these first three incubations, all 16 eggs were incubated simultaneously. In each batch, audio recordings were obtained from four isolated embryos and two pairs of contacting embryos (i.e., four contacting embryos). This yielded a total of **12 recordings from isolated embryos** and **12 from contacting embryos of the same age** across the three batches.

- **Batches 4–5:**

  In these batches, 10 eggs were initially placed in the incubator, and after 24 hours, 6 additional eggs were introduced. Audio recordings included one pair of same-age contacting embryos and three pairs of asynchronously incubated embryos (i.e., leader and follower). This yielded **4 additional recordings from same-age contacting embryos**, and **6 recordings from leader embryos** and **6 from follower embryos**.

- **Batches 6–7:**

  In the final two incubations, recordings were obtained from two isolated embryos and three asynchronous pairs per batch. This resulted in **4 additional recordings from isolated embryos**, **6 additional recordings from leader embryos**, and **6 from follower embryos**.

In total, recordings were obtained from **16 isolated embryos**, **18 contacting embryos of the same age**, **12 leader embryos**, and **12 follower embryos**. After quality control, **13 recordings from isolated embryos**, **14 from contacting embryos**, and **10 from each of the asynchronous conditions (leader and follower)** were included in the final analysis.
