# Supplementary material for: Pre-hatching social interactions mediated by acoustic signals. Dynamics of click emission and hatching synchronization in birds
Source: PLoS One. 2025 Sep 3;20(9):e0330466. doi: 10.1371/journal.pone.0330466 (PMC12407395; doi:10.1371/journal.pone.0330466)
Supplement: S2 Appendix — (PDF) [file pone.0330466.s002.pdf]

S2 Appendix. Signal acquisition and recording

Capture

Contact microphones were used for signal acquisition. These piezoelectric sensors capture vibrations from solid surfaces rather than from the air. This design serves two purposes: it enables the isolation of the targeted sound from environmental interference and provides highly sensitive recording capabilities. The microphones were secured to the eggshell using paper tape on top of the air chamber.

Amplification

Preamplifiers were constructed to amplify the amplitude of the signal by manipulating the voltage of the input signal (S1A)[1]. These preamplifiers were powered by a 5V supply obtained from the USB port of the PC used for recording the signals (S1B).

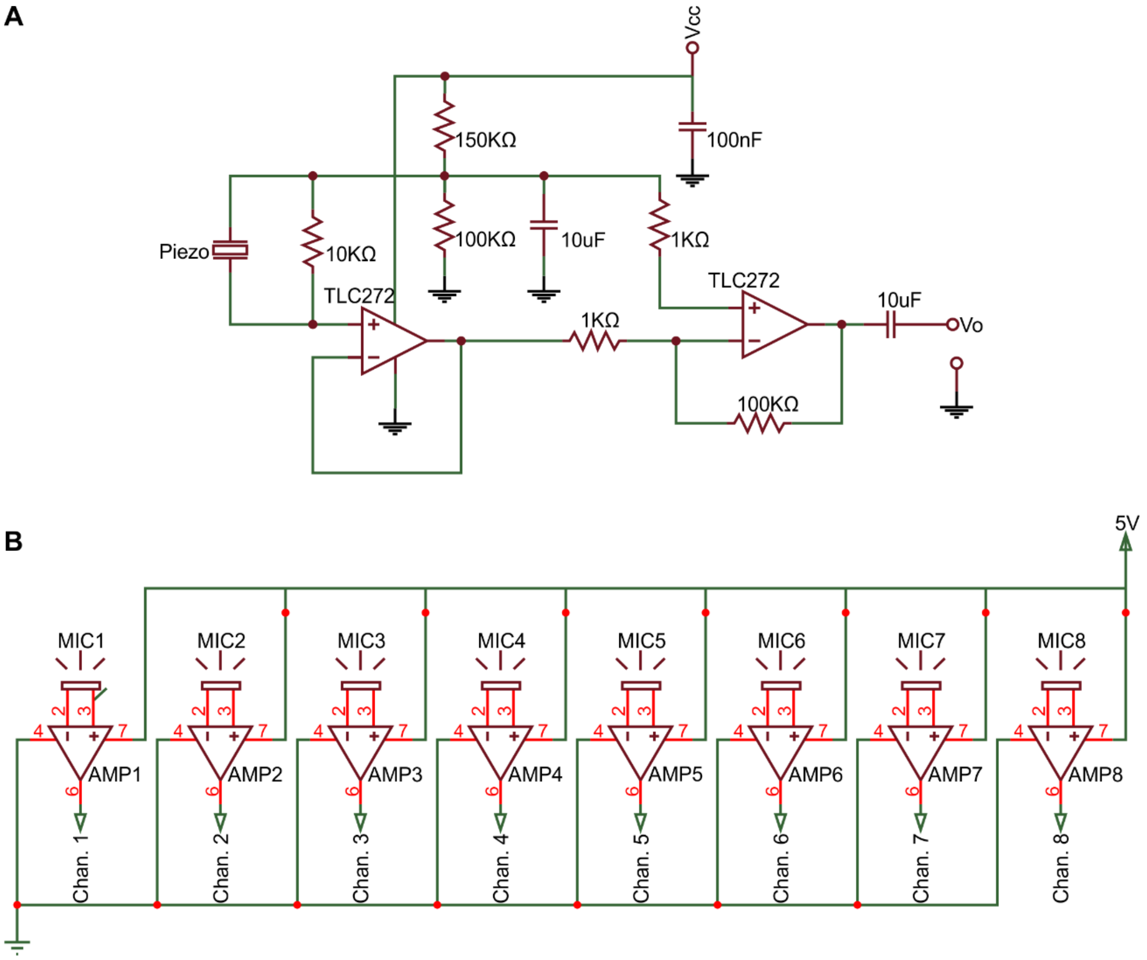

S1. Preamplifier circuit. A. Illustrates the preamplifier circuit used. **B.** Diagram of parallel power supply of the preamplifiers.

[1] Nawrath M. Laboratory for Experimental Computer Science at the Academy of Media Arts Cologne. 2012. Available from: https://interface.khm.de/index.php/lab-log/piezo-disk-preamplifier/index.html

Each channel was connected to a console (sound card) using RCA jacks.

Due to variations in signal amplitude across different recordings, and in the absence of calibrated decibel measurements, amplitude normalization was applied to allow comparison of signal dynamics over time. For each chick, the maximum amplitude recorded was set to 1, and all other values were expressed as a proportion of this maximum.

Digitization

For the simultaneous digitization of the signals, the USB8IN-SL interface was used. This interface consists of an audio card with four stereo input channels using 3.5 mm jack connectors and a USB connection that both powers the card and transmits the data to the PC. To achieve eight simultaneous tracks, four RCA-to-Plug adapters were employed, enabling the connection of two microphones per input channel.

Recording and storage

Multitrack recording was used in order to record multiple separate sound sources simultaneously, generating a .wav file for each of the inputs. The recordings were made with Adobe Audition CC software, which allows multitrack recording and is compatible with the ASIO4ALL v2 universal audio driver, used for the operation of the USB8IN-SL interface.

In order to analyze signals characteristics, the recordings were initially made with a sampling frequency of 44.1 KHz. Since this frequency exceeded the requirements, a sampling frequency of 16 KHz was established for the experiments presented in this paper.

While recordings of different durations were made, this study focuses on the analysis of 5-minute recordings taken at one-hour intervals.
