# Supplementary material for: Pre-hatching social interactions mediated by acoustic signals. Dynamics of click emission and hatching synchronization in birds
Source: PLoS One. 2025 Sep 3;20(9):e0330466. doi: 10.1371/journal.pone.0330466 (PMC12407395; doi:10.1371/journal.pone.0330466)
Supplement: S3 Appendix — (PDF) [file pone.0330466.s003.pdf]

S3 Appendix. Signal processing

The obtained audio signals were processed using MATLAB® mathematical software. Various custom software applications were created with its Graphical User Interface Design Environment (GUIDE). As a final result of the processing of each audio signal, a text file was generated containing information about the location and amplitude of each click, the sampling frequency and the duration of the signal.

Noise suppression and non-specific sound event elimination

The audio signals obtained comprised a collection of sounds emitted by the pre-hatched chicks, including claps, clicks, and peeps. Additionally, the recordings contained noise resulting from the movement of the pre-hatched chicks, ambient noise (which was of very low amplitude due to the acoustic insulation utilized during acquisition), and interference noise generated by electronic components.

In each recording, two parameters of each click were characterized: its temporal location and its amplitude. To accurately obtain these parameters, Wavelet Transform was employed to remove all components other than the clicks. Specifically, the Daubechies 12 wavelet with a decomposition into 6 levels (j) was chosen. By analyzing the details and approximations obtained from the decomposition, the following information could be observed:

- Peeps possesses high-frequency components that correspond to the frequency ranges of detail levels 1 and 2, while they are almost absent at low-frequency levels.
- Clicks are distributed across all levels of detail, but they are primarily prominent in the first four levels. The amplitude at which the clicks appear in each detail varies throughout incubation and among different eggs.
- Background noise, which includes ambient noise and interference noise, exhibits a greater amplitude in the higher details (lower frequency components).
- The noise caused by the movement of the chick is distributed in all levels of detail, with a greater amplitude in the higher levels. The signals with this type of noise, generally obtained in the last pre-hatching h, were not included in the analysis due to the inability to completely eliminate the noise, which resulted in numerous false positives.

Based on the mentioned characteristics, a semi-automatic application software, referred to as "application software 1", was developed. This software was designed to perform wavelet decomposition on the audio signals and construct a click signal based on information obtained from two specific details. These details were chosen because clicks exhibited higher amplitudes while noise and peeps had lower amplitudes. The main processes performed by “application software 1” include:

- Processing the audio signal using the Daubechies 12 wavelet transform.
- Generating a reconstructed signal by selecting and combining the two details in which clicks exhibit higher amplitudes. The user can choose these specific details.
- Locating the positions of the clicks on the reconstructed signals.
- Refining the accuracy of the click positions and amplitudes by using the original audio signal.
- Generating a final click signal that includes precise click locations and amplitudes.

“Application software 1” is used by following the steps indicated with Arabic numerals in S2.

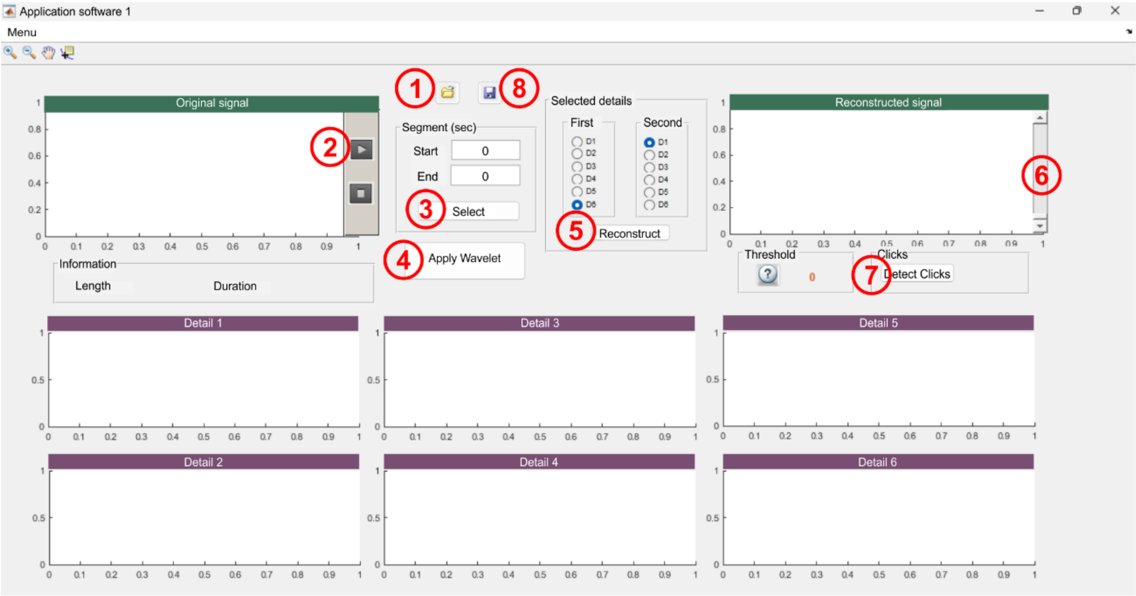

S2. Graphical interface of "application software 1" used for noise suppression, non-specific sound event elimination, and click detection. 1- Open audio signal. 2- Audio playback. 3- Selecting a segment of the audio signal. 4- Decomposition by Wavelet Transform. 5- Selection of details of interest and reconstruction of the signal. 6- Selection of the amplitude threshold. 7- Click detection. 8- Saving. Detailed description in text.

1- The "Open" function enables the user to select and load an audio file with a .wav extension as a temporary signal. The signal is then displayed in the upper left panel, where the length of the signal (expressed in the number of samples) and its duration (expressed in seconds) are indicated on the graph.

2- The "Play" function enables the user to play the loaded audio signal. By using the play command, the audio will start playing, and the user can listen to the recorded sound. The stop command can be used to halt the playback at any point.

3- The "Select" function is utilized when the user wants to analyze only a specific segment of the audio signal. The user can specify the start and end points of the desired segment, which are expressed in seconds.

4- The "Apply Wavelet" function performs a multi-resolution analysis of the audio signal using the Daubechies 12 wavelet. It decomposes the signal into 6 levels of detail and displays these details in the six lower boxes of the interface. Each level of detail corresponds to a specific frequency range:

- Detail 1: 4,000 to 8,000 kHz.
- Detail 2: 2,000 to 4,000 kHz.
- Detail 3: 1,000 to 2,000 kHz.
- Detail 4: 0,500 to 1,000 kHz.
- Detail 5: 0,250 to 0,500 kHz.
- Detail 6: 0,125 to 0,250 kHz.
- Approximation 6: 0,000 to 0,125kHz.

5- The "Reconstruct" function enables the user to select the two details in which the clicks appear with greater amplitude while the noise and peeps appear with low amplitude. It then generates a reconstructed signal using only the chosen details, disregarding the remaining details. In the

upper right panel of the interface, both the original audio signal (plotted in green) and the reconstructed signal (plotted in blue) are displayed.

6- The "Slider" function enables the user to specify the amplitude threshold for click detection. By adjusting the slider, the user can set the minimum amplitude value that a click must have in the reconstructed signal in order to be considered as such. The current value of the threshold is displayed in the "threshold" box (See sub-section Click detection).

7- The "Detect clicks" function identifies and marks the clicks present in the reconstructed signal. The detected clicks are visually represented by red circles. It is important to note that there are two versions of the “application software 1”, each with a different approach in this step.

In the version designed for analyzing records obtained from isolated pre-hatching chicks, a correction is applied (see S5). This correction involves using the original audio signal to obtain more precise values for the amplitude and location of each click. This ensures greater accuracy in the analysis of isolated pre-hatching chicks' records.

On the other hand, the version designed for analyzing records obtained from contacting pre-hatching chicks detects the location and amplitude of the clicks directly in the reconstructed signal without carrying out the aforementioned correction. Instead, the correction is performed as the last step in a second application software, which uses as input the signals generated in this application software. This second software is described in the "Elimination of transmitted clicks" section.

8- The "Save" function generates a signal that contains the temporal location and amplitude of each detected click, as well as information about the sampling frequency and length of the audio signal (see Click signals storage section).

<u>Clicks detection</u>

Click detection is performed using the MATLAB® Find Peaks tool. This algorithm enables the identification of all amplitude peaks present in the signal. Each click is represented by a matrix consisting of two parameters: the amplitude of its maximum peak and the temporal location of that value. The Find Peaks tool offers various configuration options to achieve optimal results. The following parameters were set for this analysis:

- Amplitude threshold (Minimum Peak Height): This parameter determines the minimum amplitude required for a peak in the reconstructed signal to be considered a click. It effectively filters out small peaks associated with background noise. The threshold value can be adjusted using the slider command (S2, step 6). Peaks with amplitudes below this threshold are disregarded (S3). Users can fine-tune the threshold by playing short segments of the record.

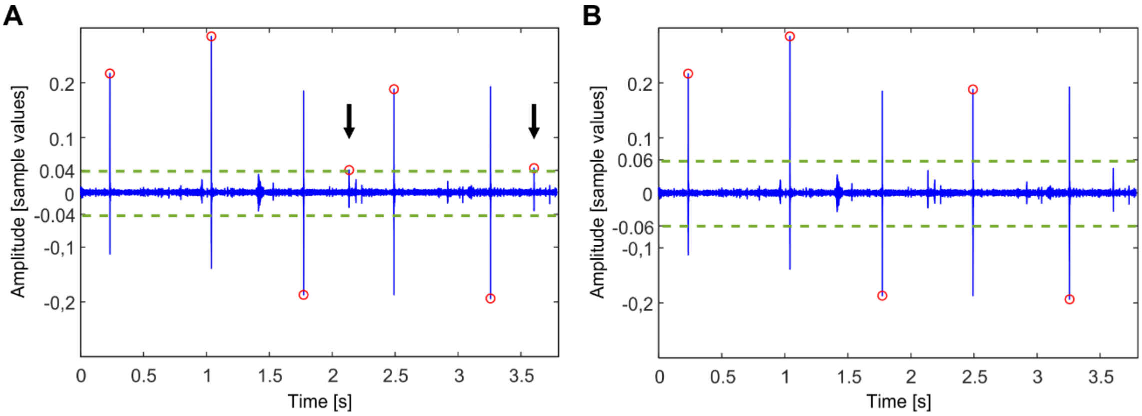

S3. Utility of the amplitude threshold parameter in the detection of peaks. A. Selected threshold = 0.04. **B.** Selected threshold = 0.06. Blue: reconstructed signal corresponding to a record made in the 6 pre-hatching hour. Red circles: detected peaks. Green dotted lines: selected threshold. False positives can be observed by choosing a low threshold (arrows in A).

- Minimum distance between peaks (MinimumPeakDistance): This parameter helps eliminate smaller peaks that appear near a larger peak. When the algorithm detects a local maximum, this parameter defines a segment around the maximum, representing the average duration of a click. Within this segment, smaller amplitude peaks are ignored (S4). This approach ensures that each click is detected only once. It is important to note that using this parameter does not pose a risk of omitting neighboring clicks. The average distance between clicks in records corresponding to periods of high click frequency (approximately 120 clicks per minute) is approximately 125 times greater than the average duration of a click (500 ms vs 4 ms, respectively).

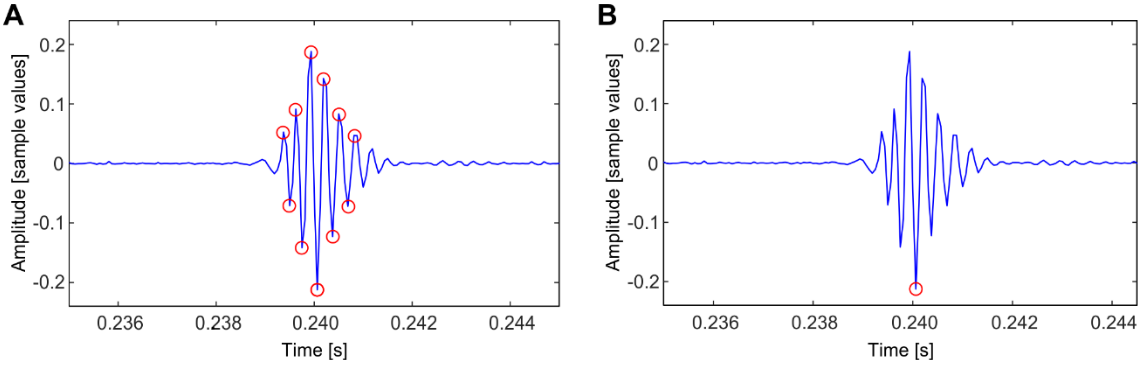

S4. Minimum distance between peaks parameter. Example of peak detection (**A**) without using the minimum distance between peaks parameter and (**B**) using this parameter. The red circles show the peaks detected. It is observed that in the case of not using this parameter, multiple peaks are detected in each click. The use of the parameter allows the detection of the peak of maximum amplitude of the click (in absolute value).

The initial detection of the click location is performed in the reconstructed signal. Using this location as an initial approximation, the original audio signal is utilized to determine the precise location and amplitude of the click. This clicks detection process involves searching for local maximum amplitudes (S5). Consequently, the temporal location of the click is redefined as the

position corresponding to the maximum amplitude in the audio record, and the amplitude of the click is determined by the maximum amplitude of the fluctuations or peaks observed in the record.

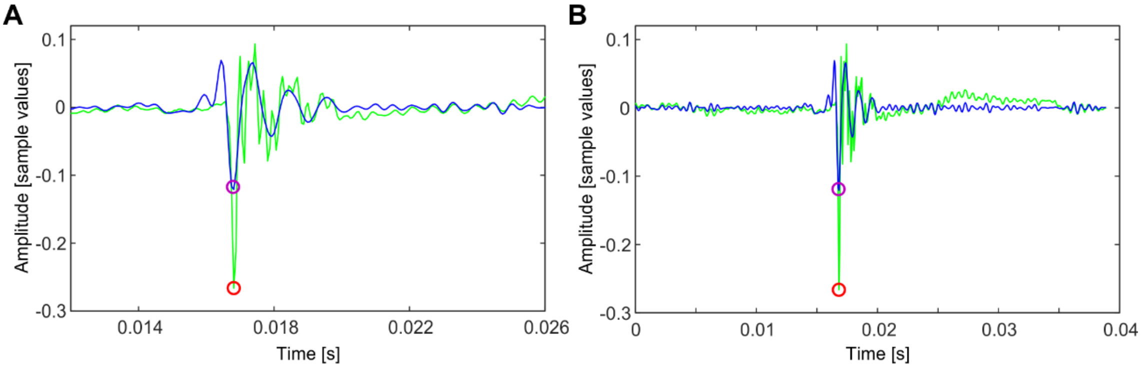

S5. Examples of the correction process for temporal location and click amplitude based on the location of the maximum click amplitude value in the original audio signal. Blue: reconstructed signal. Purple circle: highest amplitude of the click; this location and amplitude are those that are saved in the case of the software used to evaluate records made on pre-hatched chicks incubated in contact. Green: original audio signal. Red circle: maximum amplitude value of the click; this corrected location and amplitude are saved in the case of the software used to evaluate recordings made on pre-hatched chicks incubated in isolation.

S6 illustrates the results obtained by processing a signal corresponding to an isolated pre-hatching chick with “application software 1”. It can be observed that the clicks are well represented in the details of levels 1 to 5 and that they have greater amplitude in the details 3 and 4, which were selected for signal reconstruction. The upper right graph displays the reconstructed signal derived from details 3 and 4, denoted by the blue line. The green line represents the original audio signal, utilized to accurately determine the location and amplitude of the clicks.

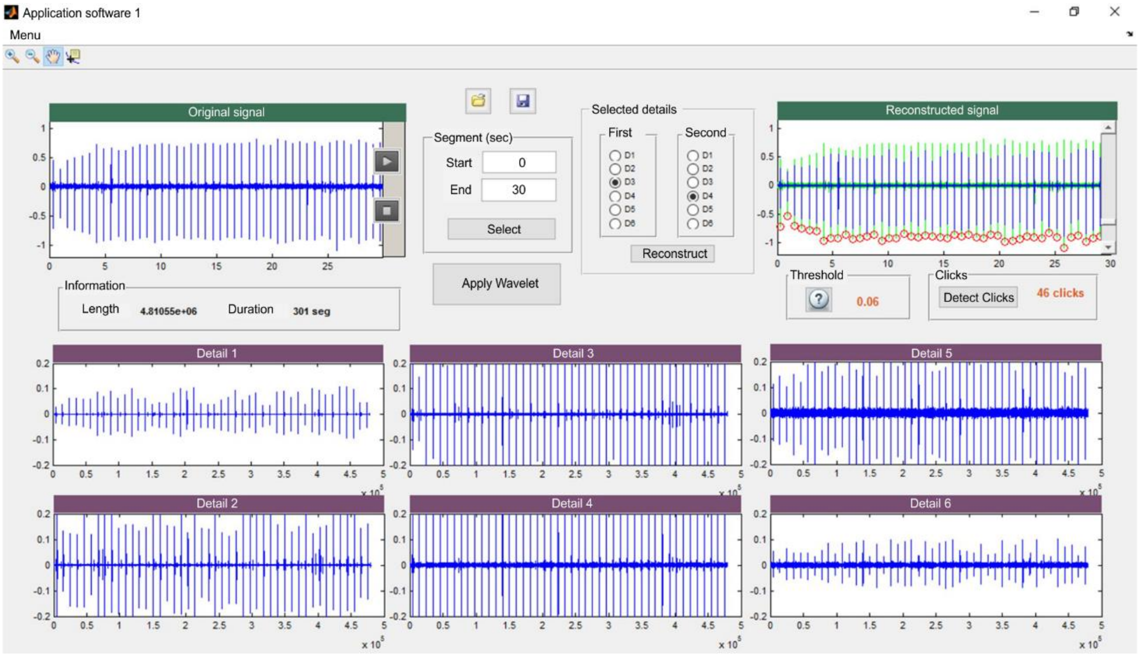

S6. Example of processing an audio signal using the “application software 1”. It shows the processing of a 30 s segment in order to appropriately visualize each click. 46 clicks were detected in this segment. Description in the text.

Elimination of transmitted clicks

The processing of records obtained from contacting pre-hatching chicks involves an additional step compared to the procedure described earlier.

In the first step, each record is processed using “application software 1”, which generates two click signals containing the location and amplitude information of all the clicks present in the reconstructed signals. These processed signals are then stored in separate text files (.txt) for each audio record.

For records obtained from contacting pre-hatching chicks, the microphone captures and records not only the clicks emitted by the pre-hatching chick from the egg it is attached to but also, to some extent, the clicks from the neighboring pre-hatching chick. However, there is significant attenuation, and the amplitude threshold used in “application software 1” manages to eliminate 94% of the transmitted clicks. Nonetheless, some clicks with high amplitude may not be eliminated by this threshold.

The second step of the processing involves distinguishing between the clicks emitted by each pre-hatching chick and those originating from the neighboring pre-hatching chick. This step allows for the individual analysis of each pre-hatching chick's behavior.

The clicking sound emitted by a pre-hatching chick is initially detected by its own microphone, and after a delay, by the microphone attached to the neighboring egg. This delay occurs due to the difference in distance that the sound must travel to reach each of the mentioned microphones. Taking into account this time delay and the previously mentioned attenuation, an application software called "application software 2" was developed. This software determines, for each pair of click signals, which click corresponds to the pre-hatching chick's own click and which comes from the neighboring pre-hatching chick.

For each click signal, the software discriminates the attenuated and delayed clicks originating from the neighboring pre-hatching chick and retains only the clicks emitted by the pre-hatching chick itself. The main processes performed by “application software 2” include:

- Identification of the clicks from each pre-hatching chick;
- Generation of two signals, one for each pre-hatching chick, containing the location and amplitude information of each click as defined in the reconstructed signals;
- Usage of the original audio signals to obtain corrected values for the precise location and amplitude of the clicks.

S7 shows the graphical interface of the “application software 2” developed and used in this second part of the processing.

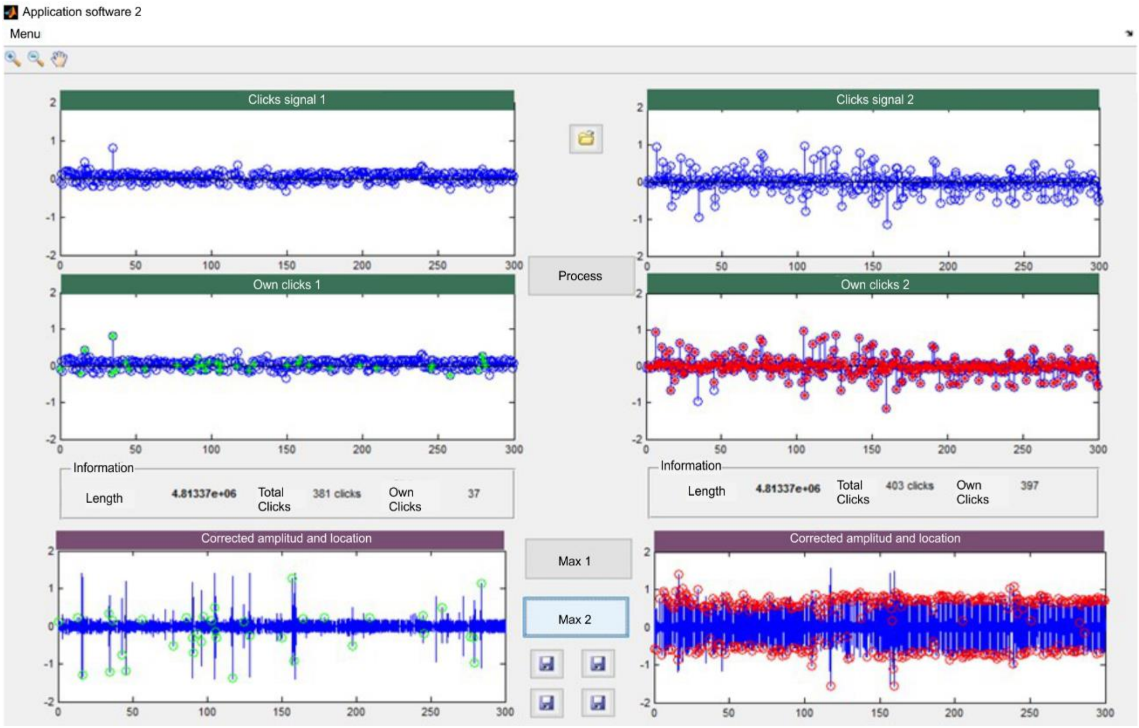

S7. Example of processing a signal using “application software 2”. Upper panels: Click signals resulting from the “application software 1” of each pre-hatching chick. When using the “application software 1” thresholds were set to allow the passage of neighboring clicks in order to illustrate the operation of this application. Middle panels: own clicks for each signal are overlapped to the original click signals. Lower panels: original segment of the audio signal used to precisely define the location and amplitude of clicks.

To use “application software 2”, the following steps must be followed:

1- Open: This function allows the user to load the text files containing the click signals obtained from “application software 1”, along with their corresponding audio files.

2- Process: In this step, the software identifies the clicks emitted by each pre-hatching chick.

3- Max 1 and Max 2: This feature corrects the location and amplitude of each click by utilizing the original audio signals. It refines the accuracy of the click parameters.

4- Save: save the new signals in .txt format. Each signal contains the temporal location and amplitude of each detected click, as well as information about the sampling frequency and length of the audio signal (see Click signals storage section).

As mentioned, in order to eliminate the transmitted clicks, an algorithm is employed that uses the click signals obtained from “application software 1”. These click signals contain information about the clicks present in both reconstructed signals. The algorithm organizes the click data into a three-column matrix, where each row represents a click. The columns are defined as follows:

- Column 1 (E): This column indicates whether the click was detected in click signal 1 or in click signal 2, denoted by a value of 1 or 2, respectively.
- Column 2 (L): It represents the precise location of the click, expressed in sample number.
- Column 3 (A): This column contains the amplitude of the click.

The matrix is then sorted temporally based on column 2 (L). This ensures that the click signals are arranged in chronological order. S8 shows an example of two click signal segments from contacting pre-hatching chicks before and after this reordering process.

	E	L	A
i=1	1	113714	-0.0372
i=2	1	126798	-0.0434
i=3	1	135264	-0.0402
i=4	2	96325	0.1291
i=5	2	110221	-0.0030
i=6	2	123957	-0.0130

	E	L	A
i=1	2	96325	0.1291
i=2	2	110221	-0.0030
i=3	1	113714	-0.0372
i=4	2	123957	-0.0130
i=5	1	126798	-0.0434
i=6	1	135264	-0.0402

S8. Ordering clicks according to their location. First three clicks of the two click signal obtained on contacting pre-hatching chicks and processed with the “application software 1”. The reassignment of positions based on the location of the clicks is illustrated.

Afterwards, the matrix is analyzed with an algorithm represented in the block diagram shown in S9.

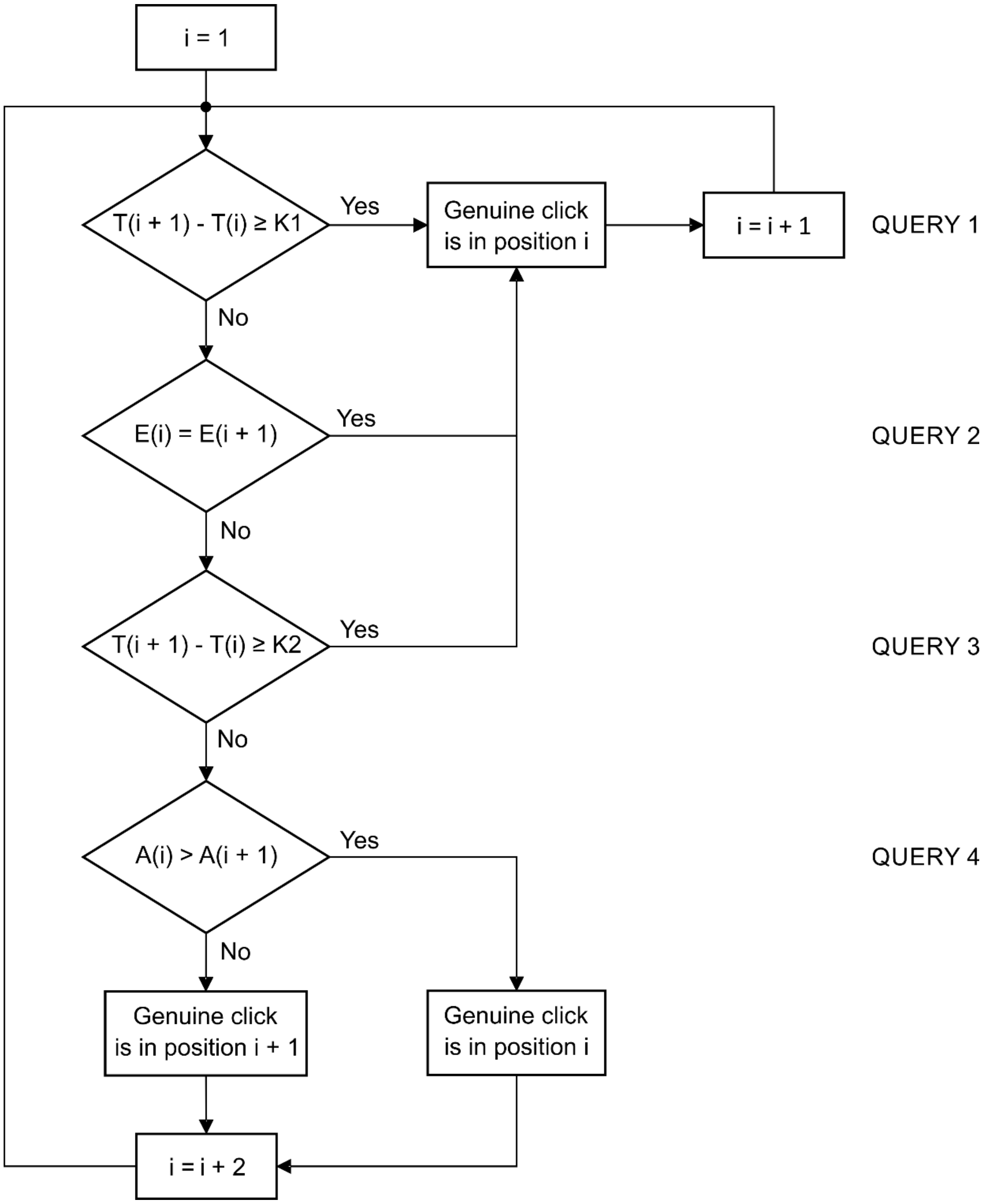

S9. Block diagram illustrating the algorithm for identifying genuine and transmitted clicks during the analysis of click signals from contacting pre-hatching chicks. K1 = 2.5 times the average duration of a click. K2 = 1/3 of the average duration of a click.

It is important to note that the chronologically ordered matrix contains both genuine clicks and transmitted clicks. The purpose of this algorithm is to retain only the genuine clicks by eliminating the transmitted clicks. Additionally, the algorithm determines to which click signal each genuine click corresponds. The algorithm begins by selecting the first click as the reference point for analysis (i=1). It then proceeds to evaluate up to four queries to determine whether the analyzed click is a genuine click or a transmitted click. After evaluating the click, the reference point is moved forward, and the next click or the second next click is selected for analysis. This iterative process continues until all available clicks have been examined and studied by the algorithm.

QUERY 1: The algorithm calculates the time until the next click. If this interval is equal to or greater than a predefined threshold value (K1), set to be 2.5 times the average duration of a click, it is determined that the click under evaluation is genuine. The algorithm then moves the reference to the next click and initiates a new cycle of analysis.

However, if the time interval is lower than threshold value, it is considered that the two clicks are close enough to each other in time to deserve further inspection. Hence, the algorithm proceeds to the next query.

QUERY 2: The algorithm compares the click signal on which the current click was detected with the click signal of the next click. If the click signals are the same, it indicates that the clicks are independent of each other, and therefore the current click corresponds to a genuine click. The algorithm then moves the reference to the next click and initiates a new cycle of analysis.

However, if the clicks signals are different, it suggests that there is a possibility that the two clicks are the same event being detected in both signals. In such cases, additional inspection is necessary, and the algorithm proceeds to the next query for further evaluation.

QUERY 3: The algorithm reevaluates the time difference mentioned in Query 1 and compares it against a different threshold (K2), which is set to be one-third of the duration of a click. If the time difference is equal to or greater than the threshold, it is determined that the click corresponds to a genuine click. As before, the algorithm continues by moving the reference to the next click, initiating a new cycle of study.

If the temporal difference is below the threshold, it is considered that the same event is being detected in both click signals. Consequently, the algorithm proceeds to the next and final query in order to establish to which click signal the genuine click corresponds and in which click signal the transmitted click must be eliminated.

QUERY 4: The algorithm compares the amplitude of the current click with the amplitude of the next click. If the amplitude of the current click is greater than the amplitude of the next click, it is determined that the current click is genuine, while the next click, which was detected shortly after the current one and on a different click signal, is a transmitted click. The algorithm then moves the reference to the second next click, as the next click needs to be eliminated, and initiates a new cycle of study.

On the other hand, if the amplitude of the next click is greater, it is considered that the genuine click is the one following the current reference click. In these cases, the duration of the transmitted click is typically very short and shorter than the genuine click. Consequently, the local maximum amplitude value of the small, transmitted click, which defines the position of that click, can precede in time, artifactually, the genuine click. Therefore, the click in position i is eliminated, and the click in position i+1 is considered a genuine click. The algorithm then moves the reference to the second next click, initiating a new cycle of study.

<u>Click signals storage</u>

The final step in processing each record, whether obtained from isolated pre-hatching chicks or from contacting pre-hatching clicks, involves storing the processed signal. The following

parameters are saved during the processing: the temporal location and amplitude of each click, the sampling frequency, and the duration of the audio signal.

This information is saved in a text file with a column structure consisting of "2*N+2" rows, where N represents the number of clicks, as specified in S10 Table.

S10. Format used to store the data

Row	Distribution
Positions [samples]	1:N
Amplitudes [samples]	N+1: (2xN)
Sample rate [Hz]	2xN+1
Audio signal length [samples]	2xN+2

The files were named using the following format: HEggNumber_Date(DD.MM)_Time(HHMM).txt. For example, a click signal generated from the record obtained from Egg 1 on February 1st at 10:35 would be named H1_01.02_1035.txt.

To facilitate further processing, an algorithm was developed to load this text file and decompose it into its constituent parts. The algorithm extracts the information from each click and creates two matrices:

1. Two-column matrix:

   Column 1: Temporal location of each click in seconds.

   Column 2: Amplitude of each click.

2. Two-column matrix:

   Column 1: Temporal location of each click in samples.

   Column 2: Amplitude of each click.

These matrices provide the necessary data for subsequent analysis and processing.
